# Venerose: a nuptial gift with implications

**DOI:** 10.1080/01677063.2025.2473095

**Published:** 2025-03-06

**Authors:** Deepanshu N. D. Singh, Matthias Soller

**Affiliations:** a School of Biosciences, College of Life and Environmental Sciences, University of Birmingham, Birmingham, UK; b Division of Molecular and Cellular Function, School of Biological Sciences, University of Manchester, Manchester, UK

**Keywords:** Cryptic female choice, Nuptial gift, Seminal fluid, Female post‑mating behaviour, Neuroendocrinal regulation

## Abstract

Males transfer many components in seminal fluid along with sperm during mating. While sex peptide is well established as a key regulator of female reproductive behaviour and success, the roles of other seminal fluid components remain less understood. A new *Drosophila* study now reveals functions for a sexually transmitted sugar in providing nutritional value and acting on nutrient-sensing neurons in the brain to maximize reproductive success. Here, we highlight the key findings of this study and explore the potential role of this sugar in male quality assessment by females and in modulation of cryptic female choice.

Offering nuptial gifts is a wide-spread strategy among animals to enhance reproductive success (Lewis & South, 2012). During courtship rituals, many birds present favoured fruits or insect prey to potential mates. Similarly, male praying mantises are often eaten after mating, which increases the size of egg cases to harbor more offspring. In *Drosophila subobscura,* males present regurgitated food from the crop to females, enhancing the fertility of those that accept it compared to those that reject it (Steele, 1986, Tanaka et al. 2017). In the ground cricket *Allonemobius socius,* males offer haemolymph as nuptial gift during copulation which improves immune function of females (Fedorka et al. 2004). Other nuptial gifts include components of the seminal fluid, such as peptides, sugars and hormones, which influence various aspects of reproductive biology (Lewis & South, 2012).

In *Drosophila melanogaster*, sex peptide (SP) transferred in the seminal fluid during mating is a key regulator of reproductive success leading to fundamental changes in female behaviour and physiology (Kubli, 2003). The SP induced post-mating response prominently includes refractoriness to mate again and induces egg laying, but SP also increases egg production, feeding, change in food choice, sleep, memory and the immune system (Hopkins & Perry, 2022). In addition, SP binds to sperm and acts as a sperm sensor (Peng et al., 2005).

SP was discovered by Pei-Shen Chen in the search for the bioactive molecule that induces the *Drosophila* post-mating response (Chen et al., 1988). During these endeavours 50 years ago they also identified a prominent sugar-like compound, 1-O-(4-O-(2-aminoethyl phosphate)-O-D-galactopyranosyl)-x-glycerol, now rediscovered by Kim et al. and termed venerose (Chen et al., 1977; Kim et al., 2024). A key step in characterizing the role of this sugar in reproductive physiology includes the identification of the biosynthetic enzymes. They found that three from the 18 *UDG-glucosyltransferase* genes with a GT28 domain led to about 50% reduction of venerose by RNA interference, but whether components of the biosynthetic pathway act redundantly or are essential for development remains to be determined.

Kim et al. then went on to describe functions for this sexually transmitted (veneral) sugar in female reproduction and sexual selection, specifically in relation to the female’s nutritional status. A first question they addressed was whether this sugar provides nutritional value. Indeed, when mated with males fed on ^32^P diet, venerose was detected in the hemolymph and phosphor was incorporated into the ovary. Moreover, females mated with males transmitting less venerose laid less eggs indicating physiological relevance for this nutritional gift.

Unlike blood-feeding mosquitos where oogenesis is induced upon feeding, *Drosophila* females produce eggs continuously (Soller et al., 1999). After mating, maximal egg production is achieved through concerted stimulation of yolk synthesis and uptake, blocking of egg resorption and stimulation of germline stem cell (GSC) proliferation (Ameku & Niwa, 2016; Soller et al., 1999).

Instructed by the difference in egg laying Young-Joon Kim and his co-workers followed up the idea that a nutrient component would be essential to stimulate egg production. Indeed, when they examined proliferation of GSCs, they found a reduction when males transmitted only half the amount of venerose. This phenotype could be rescued by injection of synthetic venerose. However, when they then tested whether venerose acts directly on ovaries, they did not observe increased GSC proliferation. Only when they cultured ovaries in the presence of adult brains, GSC proliferation was induced supporting previous observations that GSC proliferation is under endocrine control (Ameku & Niwa, 2016). This important result indicates that venerose acts via the brain.

In search of endocrine regulation of GSC proliferation, they came across diuretic hormone 44 (Dh44), which has previously been identified as a sensor for ingestion and digestion of nutrient sugars (Dus et al., 2015). When they incubated brains with venerose, six previously identified Dh44 secreting neurons responded to venerose by increased neuronal activity mediated by calcium signalling. But how could Dh44 increase GSC proliferation?

To understand this mechanism, they made use of the recent single cell gene expression data looking for Dh44 receptors in the ovary and found that *Dh44R-2*, in addition to its dominant gut expression, is also specifically expressed in terminal filament cells, that are required in the GSC niche for proliferation. Indeed, when they did RNAi knock-down of *Dh44-R2*, GSC proliferation in response to venerose was reduced. Their data further support that components from the seminal fluid can enter the circulatory system and act on the brain to influence female post mating behaviour as shown for SP (Haussmann et al., 2013; Nallasivan et al., 2021; 2024).

Kim et al. had previously found that Dh44 also extends the time females hold the ejaculate for storing sperm (Lee et al., 2015). Since venerose enhanced Dh44 activity, they speculated that venerose can increase the time females hold the ejaculate for sperm storage. Although well-nourished females did not reduce the ejaculate holding time after mating with males that had less venerose, starved females did significantly suggesting a role for venerose in sensing nutritional status of males. Moreover, when males are starved, they produced less venerose leading to a reduced ejaculate holding time and less stored sperm. Hence, as nutrient deprivation affects the quality of the male ejaculate including a reduced number of sperm (Macartney et al., 2021), their data support a role for venerose as an ejaculate quality indicator.

Female flies assess the fitness of the male and become increasingly selective after their first mating: a phenomenon linked to male quality assessment described as cryptic female choice (Eberhard, 1996). Here, females can influence offspring paternity by favouring one male’s sperm over another, but females would need to sense some value from the preferred male. The study from Kim et al. suggests that the amount of veneral sugar in the ejaculate is a quality indicator of the nutritional status of males reflecting sperm quality, but whether this sugar is modulator of cryptic female choice remains to be determined.

Doubovetzky, Billeter, and colleagues presented an elegant approach to explore female cryptic choice using transgenic males with fluorescently labelled sperm to distinguish between subsequent matings (Doubovetzky et al., 2024). Here, females bias sperm storage towards the first mate depending on levels of the male pheromones heptanal and 11-*cis*-vaccenyl acetate. A similar approach could provide valuable insights into whether females prefer males with high amounts of venerose in their ejaculate.

The data from Kim et al. further reveal a key role for a few neuroendocrine neurons in the brain in the complex quality assessment of venerose (Figure 1). This is consistent with evidence provided for SP to act through the brain in inducing the post-mating response through multiple pathways (Haussmann et al., 2013; Nallasivan et al., 2021; 2024). Likewise, SP and venerose seem to act through distinct routes suggesting a complex neuronal circuitry underlying female decision-making after mating. Recent description of the *Drosophila* neuro-connectome will be a fundamental resource to shed light on the neuronal circuitry directing such female decision making. Now with every neuron and synaptic connection in the fly brain mapped (Devineni 2024), future studies can address how females integrate multiple ejaculate-derived signals in the brain. Such research could provide a framework to distinguish between cryptic female choice and other post-mating responses.

**Figure 1.**
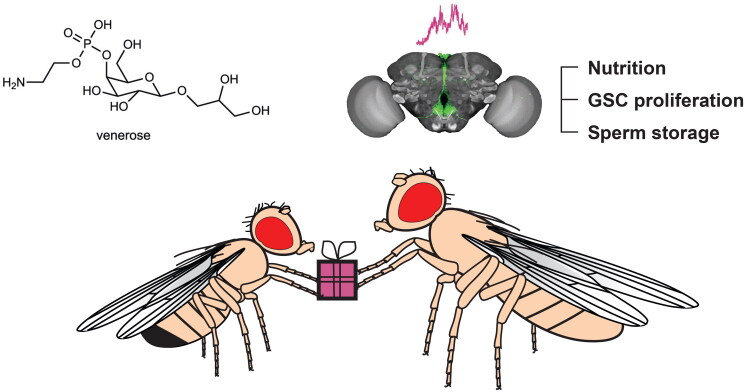
Venerose is a sexually transmitted sugar in *Drosophila melanogaster*. During mating males transfer venerose along with sperm and seminal fluid to females. Venerose is absorbed by the ovaries and serves as a nutrient source to increase egg production. Moreover, venerose enters the haemolymph and activates nutrient sensing Dh44 neurons in the brain to increase germ cell proliferation and sperm storage particularly in undernourished females.
